# The integration of psychology and medicine: an empirical study of curriculum reform from the perspective of China

**DOI:** 10.3389/fpsyg.2024.1469067

**Published:** 2024-09-04

**Authors:** Xinyan Ma, Yuze Wang, Yanmin Pu, Herui Shang, Huiqun Zhang, Xueqin Zhang

**Affiliations:** ^1^School of Health Management, Guangzhou Medical University, Guangzhou, China; ^2^School of Public Health, Guangzhou Medical University, Guangzhou, China; ^3^Practical Teaching Center, Guangzhou Medical University, Guangzhou, China

**Keywords:** new medicine, applied psychology, curriculum reform, integration of medicine and psychology, curriculum satisfaction

## Abstract

Under the background of new medicine, innovative reform of medical education is mushrooming in Mainland, China. New medicine advocates an innovative training mode supported by medicine + X discipline. In the present study, we made use of the advantages of medical colleges to reform the curriculum of applied psychology and constructed an innovative curriculum system by integrating medicine with psychology. This study aimed to evaluate the effects of the innovative curriculum system on the curriculum satisfaction of applied psychology and investigate the key factors that impact students’ curriculum satisfaction. The class of 2018–2022 college students from the Department of Applied Psychology who were under different course training systems were selected to complete the curriculum evaluation questionnaire to evaluate students’ curriculum satisfaction. The results demonstrated that curriculum satisfaction of the innovative curriculum system was significantly higher than that the one prior to the curriculum reform (*P* < 0.001), curriculum design, and teaching effect are the significant predictors affecting curriculum satisfaction. The results of this research provide innovative ideas for curriculum reform in psychology and strategies for the integration and development of medicine and psychology.

## 1 Introduction

The curriculum is highly cultural and political since it determines the vision of a society by deciding what kind of knowledge and skills are most valuable for its people and what knowledge is worth passing on (Gouëdard et al., 2020). A curriculum reflects a broader social and political agreement (Stabback, 2016), as society evolves and changes, so should the curriculum. Curriculum reform is an important and necessary measure to make students better adapt to a fast-changing world (Gouëdard et al., 2020), and curricular reform should follow the current wisdom of educational innovation and change strategy as much as possible (Reis, 2018).

In 2020, the government of China put forward a strategic initiative to expedite the innovative development of medical education which should be led by the construction of new engineering, new medicine, new liberal arts, and new agriculture, that is, “four innovations” (Wang and Ma, 2022; Hu and Wang, 2022). New medicine advocates an innovative education model supported by interdisciplinary subjects such as medicine + literature, medicine + engineering, medicine + science, and medicine + X discipline (Gu et al., 2018; Qin et al., 2022), aiming at cultivating high-level medical innovative talents who can adapt to the new generation of technological revolution represented by artificial intelligence and solve frontier problems in the medical field by using interdisciplinary knowledge (Gu et al., 2018), which coincides with the goal of interdisciplinary education (IPE) advocated by World Health Organization (2010).

According to the Center for the Advancement of Interprofessional Education (CAIPE), a key goal of IPE at the undergraduate level is to develop interprofessional knowledge, skills and attitudes that will promote interprofessional team behaviors and competence (Zheng et al., 2023a; Buring et al., 2009). Universities are increasingly offering interdisciplinary subjects and programs as an alternative to or alongside disciplinary subjects (Shay and Peseta, 2019). The glut of information and rapid spread in the contemporary world calls for students to think critically (Haapaniemi et al., 2021). Critical thinking not only helps them to sift and analyze the huge amount of information from different sources and identify the authenticity and reliability of the information but also prompts them to look at the problem from multiple perspectives, by educating integrative thinkers, schools aim to equip students with the competences necessary to connect seemingly scattered information and form a wider perspective of a whole (Haapaniemi et al., 2021; Blackshields et al., 2014). IPE transcends siloed teaching and learning approaches by emphasizing integrated learning and mutual respect between different professions in response to the new demands of health systems (Abu-Rish et al., 2012; Noll Gonçalves et al., 2021; Noll Gonçalves et al., 2023). IPE and interprofessional collaborative practice (IPCP) are intertwined movements that are reshaping educational approaches and targeted outcomes in health professional training (Lamparyk et al., 2021). It can be said that the new medicine strategy is a concrete practice of IPE.

Among the 14 disciplines in China (Ministry of Education of China, 2020b), applied psychology as a major is classified as natural science (Li Xue, 理学) (Ministry of Education of China, 2020a). In the same way, medicine is also one of the 14 disciplines. Ever since 2001, when the Ministry of Education approved the first batch of applied psychology majors in nine medical colleges in China (Yin, 2007), more than 80 medical colleges and universities have set up applied psychology majors. In view of the close relationship between medicine and psychology, as well as medical colleges and universities have exceptional advantages and resources in the field of medicine, particularly in education and teaching (Xu et al., 2015). Thus, following the “new medicine” policy and the inspiration from the medicine + X discipline concept (Gu et al., 2018; Qin et al., 2022; Yan et al., 2021), at Guangzhou Medical University, we have integrated medicine and psychology into the applied psychology curriculum to create an innovative system that reflects the unique features of a medical college.

According to Rabin et al. (2019), we focus on learner satisfaction as a learner-centered success measure (Zheng et al., 2023b), and examine the factors that impact this subjective success measure. The satisfaction of college students with the curriculum is an important factor in evaluating the success of curriculum content reform, particularly in the context of education and teaching reform (Zheng et al., 2023b). This study selected students in the context of different curriculum systems making curriculum evaluations, aimed to evaluate the effects of innovative curriculum systems on the learner satisfaction of applied psychology and identify key factors contributing to students’ curriculum satisfaction which can provide us with better support to form a long-term plan for reforming the curriculum system in new medicine context.

## 2 The context of curriculum reform of applied psychology in China

### 2.1 Existing problems in the curriculum system

Applied psychology has been launched as an independent degree program since 1993 in China (Wang et al., 2022a), it has been offered in nearly 300 colleges and universities so far (The Network Essential for College Students, 2024), one quarter of which are medical colleges and universities, which have provided talent reserves for the development of mental health services (Xu et al., 2015; Lü et al., 2020; Zhao, 2019). The “Outline of the Healthy China 2030 Plan” has put forward the objectives and requirements for the prevention and treatment of mental illness (Xinhuanet, 2016), which shows that the government attaches great importance to mental health services and also means that psychological professionals should meet higher requirements to meet the needs of society (Zhang et al., 2022). High-quality professionals are inseparable from high-level training and cultivating (Zhao et al., 2022). The specialty construction of colleges and universities always reflects the needs of economic and social development and employment most sensitively (Xu et al., 2015). However, there are still several problems in the applied psychology curriculum system (Zhao, 2019; Hang et al., 2020).

Lack of interdisciplinary systematic construction in curriculum setting. Currently, applied psychology still adopts the traditional single-discipline teaching mode. Although courses in other disciplines are integrated into the curricula, the courses in applied psychology are dominant. Moreover, in medical colleges and universities, psychology and medicine are only rigidly pieced together, which fails to reflect the deep integration of psychology and medicine (Wang et al., 2020). According to Zhang et al. (2022) the inadequacy of interaction and connection between curricula limited the expanded perspective of students’ knowledge and skills.

Lack of professional characteristics in the curriculum system. Compared with normal universities and comprehensive universities, the applied psychology major in medical colleges has a short running time, and the initial stage mostly follows the teaching mode of normal universities (Hang et al., 2020). In the curriculum system, the interdisciplinary integration between applied psychology and medicine is insufficient, applied psychology is still dominant in the curriculum system; and the value and characteristics of running a school in medical colleges are difficult to reflect compared to normal universities (Gong and Chen, 2019; Hang et al., 2020; Wang et al., 2020).

### 2.2 Medicine + X discipline talent training mode

Due to the advances in science and technology, medicine and medical technology are developing rapidly, which leads to changes in medical methods and behaviors (Gu et al., 2018), medical education around the world has changed dramatically as a result (Yan et al., 2021), which is reflected in the updating of the concepts of medical education, the innovation of medical education modes, and the optimization of the structure of medical education and so on. In order to adapt to the new round of scientific and technological revolution and industrial transformation, the new medicine is characterized by cross-integration, no longer relying on a single discipline, and integrates modern means such as big data and artificial intelligence, which has received great attention (Wang et al., 2022b).

The interdisciplinary research of “medicine + X” and the cultivation of “medicine + X” compound talents are the key contents of the new medicine construction (Yang and Wang, 2023). X discipline refers to more emerging and frontier disciplines besides medicine, including but not limited to literature, science, engineering, agriculture, biotechnology, artificial intelligence, data science, etc. The Medicine + X discipline training model promotes innovative development and progress in the medical field and realizes the complementarity and win-win situation between medicine and other disciplines facilitating the integration and application of interdisciplinary knowledge.

The development and advancement of psychology is based on medicine. At the same time, it continues to feedback and enrich the field of medicine, and the two are mutually reinforcing, forming a dynamic and mutually reinforcing cycle. Therefore, an innovative curriculum system integrating medicine and psychology has become an inevitable trend in the reform of applied psychology courses.

### 2.3 Curriculum reform integrating medicine and psychology

The curriculum reform design is student-centered and competency-based, integrating medicine and psychology to achieve teaching goals based on social demand.

**Figure 1:**
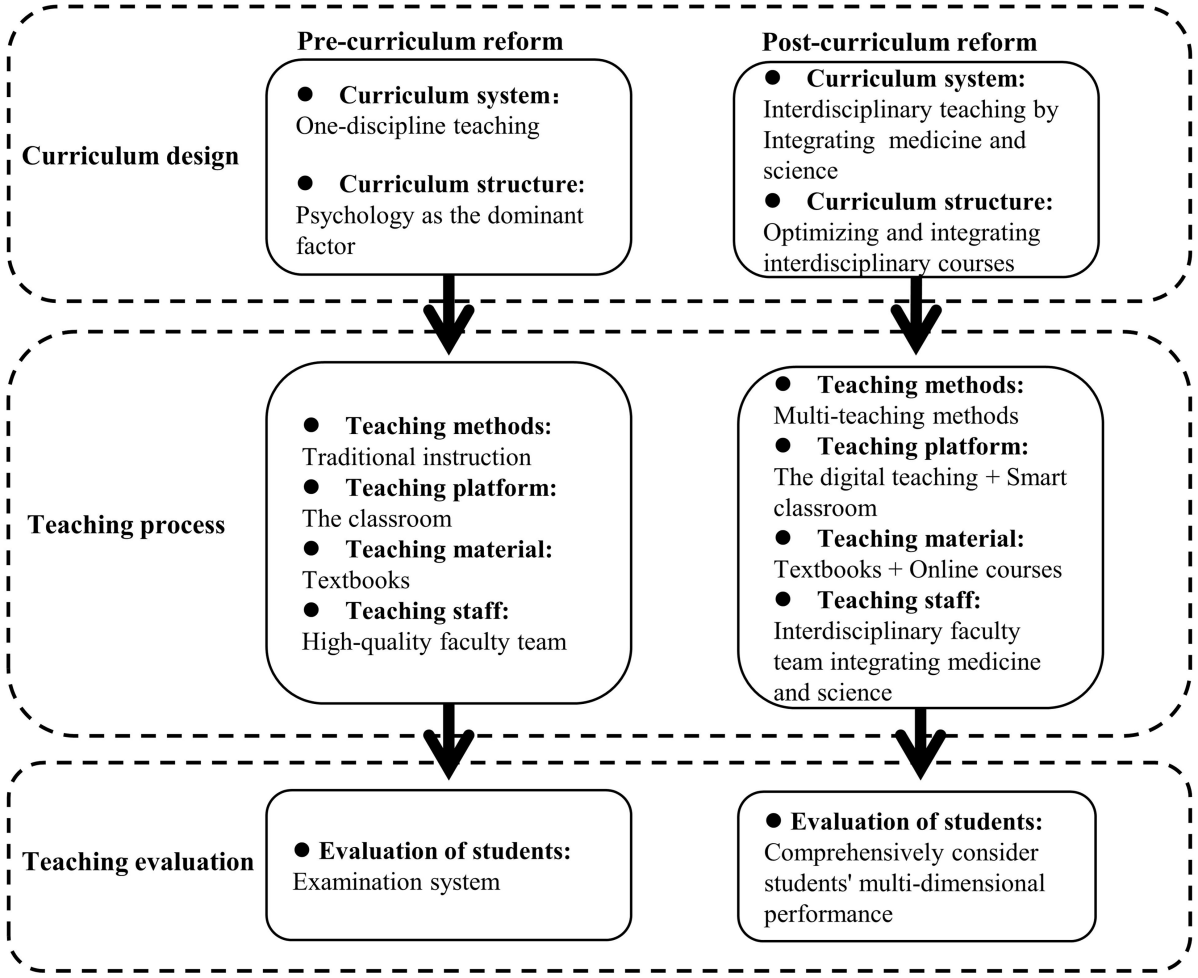
Comparison of curriculum systems.

A curriculum system for applied psychology in medical colleges has been developed based on interdisciplinary integration. As shown in Figure 1, in terms of course design, maintained the total class hours unchanged, increased the proportion of practical teaching integrating medicine and psychology, taking advantage of technologies such as big data and cloud computing to optimize the integration of medical and psychological courses, enhance the interaction and connection between courses, realizing the systematicness and integrity of the integration of medical and psychology courses, crossing the boundaries of disciplines, promoting the scientific reorganization and effective integration of medical and science courses, and reducing overlapping and duplication. In conclusion, we developed a curriculum system that embodies professional traits.

During the teaching process, our emphasis is on introducing high-quality interdisciplinary talent both from home and abroad. We encourage our teachers to practice the teaching concept of interdisciplinary integration of medicine and psychology and innovate their teaching methods. Carry out multidisciplinary integration teaching, abandon the traditional indoctrination or visiting teaching method, and give full play to students’ main role. Make full use of the new format of internet technology, build a digital teaching platform for interdisciplinary integration of medicine and psychology, develop online courses and build smart classroom to realize interdisciplinary resource sharing and promote the deepening integration of psychology and medicine by using information technology.

The advancement of curriculum should also lead to the improvement of evaluating teaching methods. A singular written test method of assessment is inadequate in fully measuring a student’s overall abilities. When it comes to evaluating teaching, a multi-dimensional assessment and evaluation system is effective because it covers various assessment contents and uses different evaluation methods. Assessment content include multi-dimensional assessments of knowledge acquisition, skills, attitudes, creativity, and teamwork abilities. Evaluation methods include Learning Management System (LMS)-based online assessment, formative assessment, program-based assessment, and interdisciplinary competency assessment. The teaching evaluation system plays an important role in improving teaching practices and promoting the development of both teachers and students. By using teaching evaluation system, we can assess students’ comprehensive ability, guide the direction of curriculum reform, and improve the overall quality of the curriculum (Zhang, 2022).

In summary, exploring and studying the innovative education model of applied psychology, which integrates medicine and psychology, and creating a multidisciplinary knowledge system for students, can benefit the applied psychology major in medical colleges. By fully integrating the psychological, spiritual, and neurological resources of medical colleges and new medical experimental technologies, it can optimize the characteristic curriculum system and content of the cross-integration of psychology and medicine. This could enhance the professional theoretical knowledge of applied psychology students, as well as their practical application ability in mental health service and psychological crisis intervention. Ultimately, these efforts will fully utilize the advantages of medical colleges.

Finally, through preliminary discussion, scientific demonstration, teaching practice, and gradual revision (Zhang et al., 2022), the research group finally constructed an innovative curriculum system integrating psychology and medicine majors.

## 3 Methods

### 3.1 Participants

The Department of Applied Psychology selected college students from the class of 2018–2022 who underwent various course training systems to complete a curriculum evaluation questionnaire. The goal was to assess the satisfaction level of the students with the curriculum. The class of 2018–2019 received training before the curriculum reform, while the class of 2020–2022 received training after the curriculum reform. Applied psychology is a four-year training system, and college students under different training programs are at cross-stage, which provides convenience for us to obtain samples.

### 3.2 Questionnaire survey

At the end of the semester, students of applied psychology from Guangzhou Medical University underwent a unified curriculum evaluation questionnaire, with the assistance of the Academic Affairs Office. This was done despite their training in different curriculum systems. The questionnaire in Supplementary Table 1 uses a five-point Likert scale with 24 items, ranging from 1 (fully inconsistent) to 5 (fully compliant). A total of 2,474 questionnaires were collected from students who evaluated 12 different courses under different training systems. Out of the total number of questionnaires, 1,268 (51.25%) were collected before the curriculum reform, while 1,206 (48.75%) were collected after the curriculum reform.

### 3.3 Statistics

The study results were analyzed and processed using SPSS25.0 software. The Mann–Whitney *U*-test was utilized to determine the disparities in students’ satisfaction with the curriculum before and after the curriculum reform. Additionally, the multiple stepwise regression analysis was employed to investigate the factors that affect course satisfaction. All the data presented above demonstrated significant differences with a *p <* 0.05.

## 4 Results

### 4.1 Descriptive statistical analysis

By using SPSS25.0, we made a descriptive statistical analysis of the results of the questionnaire, results in Table 1. The average total score of the student’s curriculum evaluation questionnaire is 103.23 ± 20.53 with a total score of 125. The average range of 24 questions is from 4.01 ± 1.09 to 4.19 ± 0.94 (out of 5). Question 2 received the highest score (4.19 ± 0.94). You think this course is of great use, and what you learn in this course will be of great help to your professional study. This shows that students have a high evaluation score for the teaching effect of the curriculum. Followed by the score of Question 1 (4.18 ± 0.94): generally speaking, you are very satisfied with the course. This indicates that the courses in applied psychology have received high levels of satisfaction from students.

**Table 1:** Curriculum evaluation questionnaire results (*N* = 2,474).

No.	AVG	*SD*	No.	AVG	*SD*
1	4.18	0.94	14	4.17	0.93
2	4.19	0.94	15	4.12	0.96
3	4.16	0.94	16	4.16	0.93
4	4.02	1.04	17	4.15	0.94
5	4.15	0.94	18	4.12	0.95
6	4.14	0.95	19	4.13	0.94
7	4.14	0.95	20	4.13	0.94
8	4.12	0.95	21	4.09	0.94
9	4.13	0.96	22	4.14	0.92
10	4.11	0.97	23	4.13	0.92
11	4.13	0.95	24	4.12	0.93
12	4.15	0.94	Total scores	103.23	20.53
13	4.15	0.93			

### 4.2 Mann–Whitney *U*-test

Mann–Whitney *U*-test is a method to test the difference between two groups of ordered variable data without specific distribution. Our questionnaire adopts a five-point scoring method, there may be data distribution deviation, so this test is adopted (MacFarland et al., 2016; McKnight and Najab, 2010). As shown in Table 2, the 12 courses include five required courses and seven optional courses. Through the Mann–Whitney *U-*test, we analyze the course satisfaction of 12 curriculums, respectively. The course satisfaction of four courses after course reform, *Criminal Psychology* (*p* < 0.05), *Social Psychology* (*p* < 0.001), *Psychological History* (*p* < 0.01), and *Biological Psychology* (*p* < 0.001), is significantly higher than before the course reform. No significant differences were found in the other eight courses. In general, however, curriculum satisfaction after the curriculum reform is significantly better than before (*p* < 0.001).

**Table 2:** Curriculum satisfaction before and after curriculum reform.

Course classification	Course name	Pre-curriculum reform	Post-curriculum reform	*z*	*p*-value
Required course	Developmental psychology	4.35 ± 0.89 (*n* = 68)	4.40 ± 0.89 (*n* = 158)	−0.358	0.721
	Social psychology	4.06 ± 0.94 (*n* = 94)	4.48 ± 0.80 (*n* = 161)	−3.617	<0.001
	Psychometrics	4.09 ± 0.94 (*n* = 68)	3.92 ± 1.12 (*n* = 74)	−0.70	0.487
	Counseling psychology	4.32 ± 0.90 (*n* = 165)	4.06 ± 1.10 (*n* = 70)	−1.489	0.136
	Biological psychology	3.87 ± 0.92 (*n* = 166)	4.30 ± 0.91 (*n* = 160)	−4.361	<0.001
Optional course	Psychological disorders and correction in children	4.18 ± 0.93 (*n* = 95)	4.35 ± 0.85 (*n* = 74)	−1.129	0.259
	Criminal psychology	3.89 ± 0.99 (*n* = 63)	4.26 ± 0.92 (*n* = 65)	−2.184	0.029
	Cognitive psychology	4.03 ± 0.93 (*n* = 39)	4.25 ± 0.91 (*n* = 73)	−1.236	0.216
	Psychological history	4.04 ± 0.98 (*n* = 164)	4.30 ± 0.90 (*n* = 257)	−2.718	0.007
	Medical psychology	4.13 ± 0.93 (*n* = 158)	4.34 ± 0.87 (*n* = 62)	−1.458	0.145
	Outline of music therapeutics	3.75 ± 1.29 (*n* = 12)	4.50 ± 0.85 (*n* = 14)	−1.631	0.103
	Human resource management psychology	4.00 ± 0.96 (*n* = 176)	3.92 ± 1.08 (*n* = 38)	−0.325	0.745
	All courses	4.08 ± 0.95 (*n* = 1,268)	4.29 ± 0.93 (*n* = 1,206)	−5.827	<0.001

### 4.3 Multiple regression analysis

#### 4.3.1 Variables selection

Based on the theoretical cognition and practice of the teaching team on curriculum reform, combined with the main points of the curriculum evaluation questionnaire, the factors affecting students’ curriculum satisfaction mainly include 23 factors in 3 aspects: curriculum design, teaching effect, and curriculum implementation. Our study focuses on identifying the primary factors that influence the satisfaction of students with their curriculum. To achieve this, we have selected Question 1 (Generally speaking, you are very satisfied with the course) from the curriculum evaluation questionnaire in Supplementary Table 1 as the dependent variable (*Y*). From Question 2 onwards, the subsequent questions (Question 2–Question 24) were designated as independent variables (*X_1_*–*X_23_*) sequentially.

#### 4.3.2 Construct model

This study has a lot of factors on curriculum satisfaction, some variables may not have significant results, and there may be some collinearity among the variables, to avoid the data estimated by the model being inconsistent with reality, the multiple stepwise regression analysis is adopted to screen the factors that affect the course satisfaction. Multiple stepwise regression analysis includes three methods: forward selection method, backward elimination method, and stepwise regression method, in which the stepwise regression method is the synthesis of the first two methods. This study adopted the backward elimination method. Firstly, a model containing all variables is established, and then the variables that contribute the least to the model are gradually eliminated. Once the variable is eliminated, it will not be considered in the next step until the significance level of the remaining variables is <0.05 (Sutter and Kalivas, 1993; Vu et al., 2015). First, we need to build a model containing all variables.

The model (1) of influencing factors of curriculum satisfaction is as follows:


(1) $$\begin{array}{l} Y=\beta_{0}+\beta_{1}X_{1}+\beta_{2}X_{2}+\beta_{3}X_{3}+\beta_{4}X_{4}+\beta_{5}X_{5}+\beta_{6}X_{6}+\\ \;\;\;\;\;\;\;\;\;\;\beta_{7}X_{7}+\beta_{8}X_{8}+\beta_{9}X_{9}+\beta_{10}X_{10}+\beta_{11}X_{11}+\beta_{12}X_{12}+\\ \;\;\;\;\;\;\;\;\;\;\beta_{13}X_{13}+\beta_{14}X_{14}+\beta_{15}X_{15}+\beta_{16}X_{16}+\beta_{17}X_{17}+\beta_{18}X_{18}+\\ \;\;\;\;\;\;\;\;\;\;\beta_{19}X_{19}+\beta_{20}X_{20}+\beta_{21}X_{21}+\beta_{22}X_{22}+\beta_{23}X_{23}+\sigma \end{array}$$

#### 4.3.3 Correlation test

Pearson correlation test is applied to verify the correlation between variables, thus laying the foundation for regression analysis. The greater the absolute value of the Pearson correlation coefficient, the greater the degree of linear correlation between variables. The correlation is very strong when the absolute value of *R* is 0.91–1.00, strong when the absolute value of *R* is 0.71–0.90, moderate when the absolute value of *R* is 0.51–0.70, weak when the absolute value of *R* is 0.31–0.50, very weak when the absolute value of *R* is 0.01–0.30, and no correlation when the absolute value of *R* is 0 (Piaw, 2008). Results are shown in Table 3, we can know that the independent variables *X*_3_ and *X*_16_ are moderately correlated with *Y*, and the other independent variables are highly correlated with *Y*.

**Table 3:** Correlation test results of variables (*N* = 2,474).

Variables	*Y*	Variables	*Y*
*Y*	1	*X_12_*	0.744^***^
*X*_1_	0.861^***^	*X_13_*	0.737^***^
*X*_2_	0.820^***^	*X_14_*	0.756^***^
*X*_3_	0.629^***^	*X_15_*	0.745^***^
*X*_4_	0.797^***^	*X_16_*	0.674^***^
*X*_5_	0.768^***^	*X_17_*	0.714^***^
*X*_6_	0.775^***^	*X_18_*	0.728^***^
*X*_7_	0.778^***^	*X_19_*	0.741^***^
*X*_8_	0.771^***^	*X_20_*	0.709^***^
*X*_9_	0.761^***^	*X_21_*	0.717^***^
*X*_10_	0.779^***^	*X_22_*	0.731^***^
*X*_11_	0.769^***^	*X_23_*	0.727^***^

^*^p < 0.05, ^**^p < 0.01, ^***^p < 0.001.

#### 4.3.4 Multiple linear regression analysis

From the above analysis, we can see that all variables have passed the correlation test, and then we need to use the backward elimination method to carry out multiple linear regression analysis.

In the first step, 23 variables were substituted into the model (1) for analysis. Then, the variable *X*_22_ with the highest *p-*value of 0.951 was eliminated, and model (2) was obtained:


(2) $$\begin{array}{l} Y=\beta_{0}+\beta_{1}X_{1}+\beta_{2}X_{2}+\beta_{3}X_{3}+\beta_{4}X_{4}+\beta_{5}X_{5}+\beta_{6}X_{6}+\\ \;\;\;\;\;\;\;\;\;\;\beta_{7}X_{7}+\beta_{8}X_{8}+\beta_{9}X_{9}+\beta_{10}X_{10}+\beta_{11}X_{11}+\beta_{12}X_{12}+\\ \;\;\;\;\;\;\;\;\;\;\beta_{13}X_{13}+\beta_{14}X_{14}+\beta_{15}X_{15}+\beta_{16}X_{16}+\beta_{17}X_{17}+\beta_{18}X_{18}+\\ \;\;\;\;\;\;\;\;\;\;\beta_{19}X_{19}+\beta_{20}X_{20}+\beta_{21}X_{21}+\beta_{23}X_{23}+\sigma \end{array}$$

In the second step, 22 variables are substituted into model (2) for analysis, and the variable *X*_21_ with the highest *p*-value of 0.774 is excluded to obtain a new model. Subsequently, the above steps were continuously carried out until step 17, when 14 variables, namely *X*_3_, *X*_5_, *X*_6_, *X*_8_, *X*_10_, *X*_11_, *X*_12_, *X*_13_, *X*_14_, *X*_15_, *X*_16_, *X_17_*, *X*_18_, and *X*_23_, were excluded, all the remaining variables had *p* < 0.05, and the results are shown in Table 4.

**Table 4:** Final results of multiple linear regression analysis.

Predictor variable	*β*	*SE*	95%CI	*t*	*p*-value	VIF	*R^2^*	*Adj-R^2^*	*F*
(constant)	0.115	0.043	(0.030–0.199)	2.663	0.008	–	0.797	0.796	1379.676***
*X_1_*	0.457	0.019	(0.420–0.493)	24.513	< 0.001	4.153			
*X_2_*	0.168	0.020	(0.129–0.208)	8.455	< 0.001	4.806			
*X_4_*	0.103	0.019	(0.065–0.141)	5.333	< 0.001	4.491			
*X_7_*	0.062	0.019	(0.024–0.100)	3.173	0.002	4.617			
*X_9_*	0.044	0.017	(0.010–0.078)	2.508	0.012	3.872			
*X_19_*	0.086	0.017	(0.053–0.120)	5.079	< 0.001	3.460			
*X_20_*	0.057	0.016	(0.026–0.088)	3.612	< 0.001	2.968			

^*^p < 0.05, ^**^p < 0.01, ^***^p < 0.001.

According to the influence coefficient and significance level of independent variables on dependent variables, it is found that *X*_1_ (*p* < 0.001), *X*_2_ (*p* < 0.001), *X*_4_ (*p* < 0.001), *X*_7_ (*p* < 0.01), *X*_9_ (*p* < 0.05), *X*_19_ (*p* < 0.001), *X*_20_ (*p* < 0.001).

After backward elimination of multiple stepwise regression analysis, the model of influencing factors of course satisfaction (3) is as follows:


(3) $$\begin{array}{l} Y=\beta_{0}+\beta_{1}X_{1}+\beta_{2}X_{2}+\beta_{4}X_{4}+\beta_{7}X_{7}+\\ \;\;\;\;\;\;\;\;\;\;\;\beta_{9}X_{9}+\beta_{19}X_{19}+\beta_{20}X_{20}+\sigma \end{array}$$

In Table 4. The *R*^2^ value is 0.797, and the adjusted *R*^2^ value is 0.796, so the fitting degree of the model is good. The *F* value of the model is 1379.676 (*p* < 0.001), which shows that the model is reliable. Multicollinearity refers to the high correlation between controlled variables. Any analysis can know the existence of multicollinearity by checking the value of the variation spread factor (VIF). When the VIF *<* 5, multicollinearity is not serious. If VIF > 5, multicollinearity is significant. When the VIF > 10, multicollinearity will be more serious (Ghani et al., 2010). In our study, all VIF values are below 5, which means that there is no significant multicollinearity between independent variables. Finally, the multiple linear regression model of influencing factors of course satisfaction is obtained:


$$\begin{array}{l} Y=0.115+0.457X_{1}+0.168X_{2}+0.103X_{4}+\\ 0.062X_{7}+0.044X_{9}+0.086X_{19}+0.057X_{20}+\sigma \end{array}$$

## 5 Discussion

The average score of students’ satisfaction with courses is 4.18 ± 0.94. Generally speaking, students’ satisfaction with courses in applied psychology is at a high level. Through the Mann–Whitney *U*-test of the overall curriculum satisfaction before and after the curriculum reform, it is found that curriculum satisfaction after the curriculum reform is significantly higher than that before the curriculum reform (*P <* 0.001). At the same time, we also analyzed the differences between the 12 courses before and after the reform, which found that the 4 courses’ teaching satisfaction after the curriculum reform was significantly higher than that after the curriculum reform. The course satisfaction of four courses, *Criminal Psychology* (*p* < 0.05), *Social Psychology* (*p* < 0.001), *Psychological History* (*p* < 0.01), and *Biological Psychology* (*p* < 0.001), is significantly higher than before the course reform, which may be because all these four courses are interdisciplinary, which can broaden students’ disciplinary horizons and enrich multidisciplinary knowledge and skills. Moreover, according to Zeff (2007), the utilization of diversified teaching methods gives learners various ways of acquiring information and knowledge that enables students to obtain a positive learning experience and enhance their cognition and understanding in the area of psychology whereas the differences in the remaining 8 courses are not significant.

In general, students’ satisfaction with the courses of applied psychology specialty after the curriculum reform is higher than that before the curriculum reform, but it is not balanced in the evaluation of the satisfaction of specific courses, which also exposes the problems of insufficient curriculum systematization in the innovative education mode of medicine science integration of applied psychology specialty and insufficient penetration and integration of innovative reform measures in all professional courses.

Through the construction of a stepwise regression model, it is found that the influencing factors of students’ satisfaction with curriculum evaluation are in descending order: (1) whether the course is useful and whether the things learned in the course will be of great help to students’ professional learning; (2) whether the teaching objectives of the course are clear, and whether the requirements and expectations of the course for students are clear; (3) whether the semester of the course is reasonable; (4) whether the course examination improves students’ understanding of the course’s core concepts; (5) whether students can understand the relationship between the topics (chapters) of the course; (6) whether the learning of the course can enable students to master the knowledge, theory, and skills of the course; (7) course teaching has well stimulated your interest in learning and mobilized your enthusiasm for learning. The coefficient of influence of the above seven factors on course satisfaction is 45.7, 16.8, 10.3, 8.6, 6.2, 5.7, and 4.4% in turn.

Analyzing these factors, we think that curriculum design and teaching effect will have a positive impact on students’ curriculum satisfaction. A clear teaching objective of the course affects curriculum satisfaction by 16.8%. According to Soulie and Cosson (2021), a goal-oriented approach to teaching proved essential to determine the overall structure of the teaching program, as well as the content of specific curriculums, and the nature of the examinations. In addition, keeping in mind the ultimate goal is also conducive for students to separate useful knowledge from useless knowledge. Having mastered a certain amount of knowledge, theory, and skills in the course study and what you have learned will be of great help to your professional study with the influence degrees of 5.7 and 45.7%, respectively, on curriculum satisfaction. A study pointed out that learning self-efficacy can promote students’ mastery of knowledge and skills (Zheng et al., 2023b). The higher the sense of self-efficacy, the higher the degree of knowledge acceptance of the course content, and the more knowledge they can understand and accept. Correspondingly, curriculum satisfaction will be improved. The course teaches stimulates students’ interest and enthusiasm for learning, which shows that teaching innovation makes students feel “pleasantly surprised” (Mayhew et al., 2021), and new teaching approaches were applied to get rid of traditional ideas and existing procedures (Cao et al., 2022), therefore students have a pleasant learning experience and enjoy the classroom, with an influence of 4.4% on curriculum satisfaction. The term of the course is reasonable, that is, the course content conforms to the student’s professional knowledge background at the corresponding learning stage, and the chapters of the course content are closely connected and reasonable, which can reduce the students’ understanding cost, reduce the learning difficulty and improve the learning effect to a certain extent. That is consistent with Oliver et al. (2008), the context of learning should be closely connected which can promote students to better internalize what they have learned and improve their teaching satisfaction, with the influence degrees of 10.3 and 6.2%, respectively. Reasonably designed course examinations can deepen students’ understanding of core concepts. As Leung et al. (2008) mentioned it is also necessary to forcibly memorize some knowledge so that students can build their knowledge framework, thus better consolidating the teaching effect and improving their learning experience and satisfaction, with an impact of 8.6%.

## 6 Conclusion and limitation

### 6.1 Conclusion

This study assessed the effectiveness of an innovative curriculum system based on the integration of medicine and psychology in the context of the new medical science, using learner satisfaction as the main indicator and exploring its influencing factors. In conclusion, curriculum design is the basis of teaching, and scientific curriculum design can help teachers organize teaching activities effectively and improve the teaching effect. The teaching mode of the integration of medicine and psychology promotes students’ active participation and interest in learning and improves the teaching effect and satisfaction by clarifying the teaching objectives and contents, rationally planning the teaching progress and forms, and choosing appropriate diversified teaching methods and multi-dimensional evaluation methods. It is still necessary to strengthen the systematic construction of curriculum reform, deeply integrate and infiltrate curriculum innovation and reform measures, and promote the comprehensive and balanced development of professional courses.

### 6.2 Limitations of this study and future work

The questionnaire is a curriculum evaluation questionnaire for all majors in university. Although it can reflect some problems in curriculum reform, it is not a targeted investigation of the education mode of the integration of medicine and psychology for applied psychology majors, and it fails to reflect more comprehensive and in-depth issues.

Curriculum innovation should lead to the innovation of curriculum evaluation. Given the specific measures of education mode of applied psychology, we should formulate targeted curriculum evaluation questionnaires, innovate and expand the methods of curriculum evaluation, expand the survey objects, and formulate multi-dimensional curriculum evaluation questionnaires for students and teachers, respectively.

With the development of social economy and culture, the enrichment of subject fields, and the expansion of subject frontiers, the continuous improvement and optimization of the curriculum system requires a longer-term plan to carry out. As this study is a cross-sectional study, the long-term and short-term longitudinal study can be used to continuously evaluate and evaluate curriculum satisfaction, and adjust the strategies and plans of curriculum reform in time, to promote the rapid development of the discipline.

## Data Availability

The raw data supporting the conclusions of this article will be made available by the authors, without undue reservation.
