# Supplementary material for: The integration of psychology and medicine: an empirical study of curriculum reform from the perspective of China
Source: Front Psychol. 2024 Sep 4;15:1469067. doi: 10.3389/fpsyg.2024.1469067 (PMC11408292; doi:10.3389/fpsyg.2024.1469067)
Supplement: Supplementary file 1 [file Table_1.DOCX]

| Supplementary Material Supplementary Table **Supplementary Table 1.** Curriculum evaluation questionnaire | | |
| --- | --- | --- |
| No. | | Questions |
| 1 | Generally speaking, you are very satisfied with the course | |
| 2 | You think this course is very useful, and what you learn in this course will be of great help to your professional study. | |
| 3 | You are well aware of the requirements and expectations of this course. | |
| 4 | Teaching the content of this course is challenging, and you must study hard to meet the requirements. | |
| 5 | The semester of this course is reasonable. | |
| 6 | The class hours of this course are reasonable. | |
| 7 | The teaching arrangement of this course is systematic and logical. | |
| 8 | You can understand the relationship between the topics (chapters) of the course. | |
| 9 | The class time of this course has been used efficiently. | |
| 10 | This course has well stimulated your interest in learning and mobilized your enthusiasm for learning. | |
| 11 | Teachers can clearly explain the important and difficult points of the course and answer questions for students after class. | |
| 12 | Teachers encourage students to ask questions and express their opinions freely. | |
| 13 | Teachers make better use of network-assisted teaching. | |
| 14 | Teachers attach importance to students' cooperative learning. | |
| 15 | Teachers can adjust and improve teaching according to your learning situation. | |
| 16 | Learning resources provided by teachers are of great help to your study. | |
| 17 | You are well aware of the evaluation method of this course. | |
| 18 | The teacher can give timely and valuable feedback to help you improve your study. | |
| 19 | Course examination can let you know your mastery degree of the course. | |
| 20 | Course examination improves your understanding of the core concepts of the course. | |
| 21 | Learning the course enables you to understand and master the knowledge, theory, and skills of the course. | |
| 22 | Learning the course can improve your professional quality and guide you to establish a correct world outlook, outlook on life, and values. | |
| 23 | Learning the course improves your critical thinking and problem-solving ability. | |
| 24 | Learning the course cultivates your creativity and innovation ability. | |
